# HPV infection and cervical neoplasia: associated risk factors

**DOI:** 10.1186/s13027-015-0011-3

**Published:** 2015-05-26

**Authors:** Andrea Alves Ribeiro, Maria Cecília Costa, Rosane Ribeiro Figueiredo Alves, Luísa Lina Villa, Vera Aparecida Saddi, Megmar Aparecida dos Santos Carneiro, Luiz Carlos Zeferino, Sílvia Helena Rabelo-Santos

**Affiliations:** Institute of Tropical Pathology and Public Health, Federal University of Goiás, Goiânia, GO Brazil; Santa Casa de São Paulo, INCT-HPV at Santa Casa Research Institute, School of Medicine, São Paulo, Brazil; Department of Radiology and Oncology, School of Medicine, University of São Paulo and Cancer Institute of the State of São Paulo, ICESP, São Paulo, Brazil; Santa Casa de Misericórdia de Goiânia, Goiânia, GO Brazil; Pontifical Catholic University of Goiás, Goiânia, GO Brazil; School of Medicine, Federal University of Goiás, Goiânia, GO Brazil; Program in Environmental Sciences and Health, Pontifical Catholic University of Goiás, Goiânia, GO Brazil; Laboratory of Oncogenetics and Radiology, Associação de Combate ao Câncer, Goiás, Goiânia, GO Brazil; State University of Campinas (UNICAMP), Campinas, SP Brazil; School of Pharmacy, Federal University of Goiás, Goiânia, GO Brazil

**Keywords:** Human papillomavirus, Risk factors, Abnormal cervical smear

## Abstract

**Background:**

Behavioral risks such as age at first sexual intercourse, number of sexual partners and partner’s sexual behavior are associated with an increased risk of HPV infection, persistence of the infection and the development of neoplastic precursor lesions. The objective of this study was to evaluate the risk factors associated with HPV positivity and with a diagnosis of cervical neoplasia in women referred with an abnormal cervical smear.

**Methods:**

This study evaluated a series of 198 women referred with an abnormal cervical smear. Risk factors for HPV infection were investigated using a questionnaire. All cervical specimens were tested for 27 HPV genotypes using the Roche polymerase chain reaction reverse line blot assay.

**Results:**

The overall prevalence of HPV was 87 %. First sexual intercourse before 16 years of age was significantly associated with a positive HPV test (OR 4.41; 95 %CI: 1.20 − 19.33; *p* = 0.01). A significant association was also found between this risk factor and CIN 1 lesions or worse (OR 2.2; 95 %CI 0.94 − 5.08; *p* = 0.03).

**Conclusions:**

The age at which a woman begins to be sexually active is associated with HPV infection and with a diagnosis of cervical neoplasia.

## Background

Human papillomavirus (HPV) infection of the genital tract is thought to be the most common sexually transmitted virus. The prevalence of this infection is age-dependent and is higher in women between 15 and 25 years of age. The decrease in the rate of HPV infection with increasing age likely results from some combination of decreased HPV exposure, the self-limiting nature of most infections and a resistance to re-infection [1, 2].

Persistent infection with human papillomavirus (HPV) is a prerequisite for cervical cancer and its precursor lesions [3, 4]. The higher risk of HPV infection among younger women has been related to a lack of adaptive immune responses and/or the relatively larger area of cervical epithelium undergoing squamous metaplasia [5]. Structurally, the adolescent cervix is different from that of adults in that it has greater areas of immaturity, described as a predominance of columnar and metaplastic epithelium. An example of the fragility of this area is the common presence of blood when cervical smears are obtained in adolescents who have large areas of ectopy [6, 7].

Behavioral risks such as age at first sexual intercourse, the number of sexual partners and the partner’s sexual behavior are associated with an increased risk of HPV infection [8, 9]. Studies have shown that higher prevalence rates of high-risk HPV infection and also a higher proportion of women with multiple high-risk infections may be related to sexual behavior, social class, high parity, lack of barrier contraceptive protection and long-term use of oral contraceptives [10].

Co-factors that would act by affecting the way in which HPV is acquired, its persistence and development, as well as its progression to neoplastic lesions, are common [11]. In addition to persistent HPV infection with high-risk genotypes, multiple sexual partners, overall lifetime number of partners, high parity, the use of oral contraceptives and smoking are risk factors associated with the development of cervical cancer [7, 5, 12].

Therefore, the objective of this study was to evaluate the risk factors associated with HPV infection and with neoplastic diagnoses in women referred with an abnormal cervical smear.

## Methods

### Specimens

This study was conducted at a colposcopy referral clinic in Goiânia, central Brazil. Between January 2006 and March 2009, a series of 198 women with an abnormal cervical smear were included. The study protocol was approved by the Internal Review Board of the Pontifical Catholic University of Goiás with the registration CEP 003/05.

All the participants answered a complete standardized questionnaire concerning age, smoking, age at first sexual intercourse, number of lifetime sexual partners, oral contraceptive use and number of pregnancies. Exclusion criteria consisted of pregnancy and clinical signs of immunosuppression. The study protocol was approved by the institute’s internal review board and all patients signed an informed consent form.

At colposcopy, a cervical sample was taken for a second conventional smear using a cervical brush, and the residual material was rinsed with and then stored in 1.0 mL Universal Collection Medium (QIAGEN Sample & Assay Technologies, São Paulo, Brazil) for HPV DNA testing. Biopsies were taken from any colposcopically abnormal area. Women with a suspicious image penetrating the cervical canal and those in whom colposcopy was unsatisfactory and a second cervical smear was abnormal were submitted to cervical conization. Women with invasive carcinomas were treated in compliance with the appropriate clinical guidelines. When a woman was submitted to more than one histological examination, the most severe diagnosis was the one taken into consideration.

During the study period, 193 biopsies were performed and analyzed according to the criteria defined by the World Health Organization, being classified as normal/cervicitis, CIN 1, CIN 2, CIN 3, invasive squamous cell carcinoma (SCC) or invasive adenocarcinoma. The remaining 5 women in the study tested negative at colposcopy and negative in their repeat cervical smear; therefore, the final diagnosis in these cases was negative for neoplasia. For the purposes of analysis, they were classified together with the women with normal results at histology or those with a finding of cervicitis. These 5 women were scheduled to return for follow-up visits every 3 months, and at the cut-off date for this analysis the duration of follow up in these cases ranged from 3 months to 2 years.

### Sample processing, DNA extraction and HPV-DNA testing

The samples were processed as previously described [13]. HPV amplification and genotyping was carried out by using the Roche polymerase chain reaction (PCR)-based Linear Array® HPV genotyping test (Roche Molecular Systems, Branchburg, NJ, USA). HPV DNA was amplified using the L1 consensus biotinylated PGMY09/PGMY11 primer set in a thermal cycler at 95 °C for 13 min, followed by desnaturation for 1 min at 95 °C, annealing for 1 min at 55 °C, and extension at 72 °C for 1 min for a total of 40 cycles. Amplification was followed by a 5-min terminal extension step at 72 °C. Biotinylated GH20 and PC04 primers to the β-globin gene were used as amplification control. PCR products were denatured in 1.6 % sodium hydroxide (NaOH) and hybridized to an immobilized probe array containing probes for β-globin at 2 concentrations plus 37 HPV genotypes. One of groups included the HPV types 16, 18, 26, 31, 33, 35, 39, 45, 51, 52, 53, 56, 58, 59, 66, 68, 69, 73 and 82. The other group included the HPV types 6, 11, 40, 42, 54, 55, 61, 62, 64, 67, 70, 71, 72, 81, 83, 84, IS39 and CP6108. Positive hybridization was detected by streptavidin-horseradish peroxidase-mediated color precipitation on the membrane at the probe array.

According to their epidemiological association with cervical cancer and consolidated by biological studies, 12 types of HPV (HPV-16, HPV-18, HPV-31, HPV-33, HPV-35, HPV-39, HPV-45, HPV-51, HPV-52, HPV-56, HPV-58 and HPV-59) have now been consistently classified as hrHPV (also known as IARC class I). HPV-68 has been classified as probable high-risk (also known as IARC class 2A), and another seven types have been classified as possible high-risk (HPV-26, HPV-53, HPV-66, HPV-67, HPV-70, HPV-73 and HPV-82; also known as IARC class 2B) [14, 15].

### Histopathology

The specimens were reviewed in accordance with the World Health Organization criteria (Scully et al. 1994) [16] and were classified as: CIN 1, CIN 2, CIN 3, invasive squamous carcinoma or invasive adenocarcinoma.

### Statistical analysis

A database including demographic and behavioral factors was analyzed using the SPSS statistical software package (SPSS, Inc., Chicago, IL, USA). Crude and adjusted odds ratios (OR) for trends with 95 % confidence intervals (95 % CI) were calculated and the chi-square test was used whenever appropriate. The significance level for the tests (*p*) was set at 0.05.

## Results

The demographic, sexual and reproductive characteristics of the study sample are shown in Table 1. The mean age of the women referred with abnormal cervical smears was 34 years, with 56.1 % (111/198) being < 35 years of age and 43.9 % (87/198) being ≥ 35 years of age.

**Table 1:** Demographic variables of the women enrolled in this study

Age	n	%
< 35 years	111	53.1
≥ 35 years	87	43.9
Initiation of sexual activity		
<16 years	64	32.3
≥16 years	134	67.7
Number of partners		
1	40	20.2
≥2	158	79.8
Pregnancies		
None	31	15.7
≥1	167	84.3
Oral contraceptive use		
Yes	49	24.7
No	149	75.3
Smoking		
Yes	42	21.2
No	156	78.8
HPV infection		
Yes	171	86.4
No	27	13.6
HPV 16 and 18		
Yes	84	42.4
No	87	43.9
Other HPV types		
Yes	87	43.9
No	84	42.4
Total	198	100

Overall, 32.3 % of the women (64/198) had their first sexual intercourse prior to 16 years of age. Forty of the women (20.2 %) had had a single sexual partner during their lifetime, while 158 (79.8 %) reported having had two or more partners over their lifetime. Most women (167/198, 84.3 %) reported having been pregnant at least once. Oral contraceptive use was reported by 49 women (24.7 %). Forty-two women (21.2 %) reported smoking. Of the women referred with abnormal cervical smears, 87 % (171/198) had an HPV infection, including 42.4 % of women infected with HPV types 16 and 18 and 43.9 % of women infected with other HPV types.

The distribution of HPV genotypes and histological diagnosis is shown in Table 2. The prevalence of HPV 16 and 18 in the women with a histological diagnosis of CIN 1 was 31.8 % (21/66). With respect to more severe lesions, HPV 16 and 18 infections were present in 61.5 % of cases (24/39) of CIN 2, 43.2 % of cases (19/44) of CIN 3 and 88.9 % of cases (8/9) of invasive carcinoma (including 5 squamous cell carcinomas and 3 adenocarcinomas).

**Table 2:** Prevalence of HPV genotypes and histological diagnosis

HPV types	Negative	CIN1	CIN2	CIN3	Invasive ^a^	Total
	n (%)	n (%)	n (%)	n (%)	n (%)	n (%)
Other HPV types	18 (45)	35 (53.0)	12 (30.8)	21(47.7)	1(11.1)	87 (43.9)
HPV 16/18- positive	12 (30)	21 (31.8)	24 (61.5)	19 (43.2)	8 (88.9)	84 (42.4)
HPV-negative	10 (25)	10 (15.2)	3 (7.7)	4 (9.1)	0	27 (13.6)
Total HPV positive	30 (75)	56 (84.8)	36 (92.3)	40 (90.9)	9 (100)	171 86.4)
Total	40 (100)	66 (100)	39 (100)	44 (100)	9 (100)	198 (100)

^a^Invasive carcinoma included 3 cases of adenocarcinomas. HPV: Human papillomavirus

A significant association was found among women who had their first sexual intercourse prior to 16 years of age and HPV infection (OR 4.41; 95 %CI: 1.20- 19.33). In addition, a positive association was found between a woman having initiated her sexual life prior to 16 years of age and infection by HPV types 16 and 18 (OR 4.68; 95 %CI: 1.20 - 21.32). The same analysis was performed with respect to these risk factors and other types of HPV; however, no such association was found. Furthermore, no association was found between any of the other risk factors (such as age, number of sexual partners, number of pregnancies, oral contraceptive use and smoking) and HPV infection (Table 3).

**Table 3:** Association between HPV infection and variables associated with clinical behavior

Risk factor	OR (95 % CI)^a^	*p*-value	OR (95%CI)^b^	*p*-value	OR (95%CI)^c^	*p*-value
Age						
<35 years	0.86(0.35-2.10)	0.71	0.87(0.33-2.29)	0.76	0.97(0.51-1.85)	0.02
≥35 years						
Initiation of sexual activity						
<16 years	4.41(1.20-19.33)	0.01	4.68(1.20-21.32)	0.01	0.9(0.46-1.76)	0.7
≥16 years						
Number of partners						
1	0.8(0.32-1.97)	0.3	1.2(0.37-3.88)	0.7	0.9(0.40-2.04)	0.8
≥2						
Number of partners						
<3	1.21(0.5-2.95)	0.6	1.2(0.45-3.08)	0.7	1.06(0.55-2.05)	0.85
≥3						
Pregnancies						
None	0.64(0.14-2.45)	0.48	0.7(0.15-3.32)	0.67	0.7(0.3-1.80)	0.46
≥1						
Oral contraceptive						
Yes	1.1(0.38-2.93)	0.88	1.49(0.48-4.57)	0.44	0.5(0.25-1.19)	0.09
No						
Smoking						
Yes	1.6(0.50-5.99)	0.38	1.8(0.50-6.97)	0.32	0.83(0.38-1.82)	0.62
No						

^**a**^confidence interval. HPV: Human papillomavirus

^b^HPV types 16 and 18, Reference HPV-negative

^c^Other HPV types except HPV 16 and 18, Reference HPV 16 and 18

A statistically significant association was also found between a histological diagnosis of cervical intraepithelial neoplasia (CIN) 1 or worse and women who had had their first sexual intercourse prior to 16 years of age (OR 2.2; 95 %CI: 0.94 - 5.08). CIN 1 or worse was also significantly associated with HPV infection (OR 2.76; 95 %CI: 1.05 - 7.19) and with HPV 16 and 18 infections (OR 3.53; 95 %CI: 1.17 - 10.67). However, no association was found with any of the other HPV types (OR 2.25; 95 %CI 0.80 - 6.36) (Table 4).

**Table 4:** Risk factors and association with a histological diagnosis of CIN 1 or worse

Risk factor	OR (95%CI)	*p*-value
Age		
<35 years	1.19 (0.59-2.40)	0.3
≥35 years		
Initiation of sexual activity		
<16 years	2.2 (0.94-5.08)	0.03
≥16 years		
Number of partners		
1	0.7 (0.36-1.54)	0.2
≥2		
Number of partners		
<3	1.21 (0.5-2.95)	0.6
≥3		
Pregnancies		
None	0.46 (0.18-1.77)	0.06
≥1		
Oral contraceptive use		
Yes	1.2 (0.5-2.62)	0.3
No		
Smoking		
Yes	0.6 (0.28-1.22)	0.11
No		
HPV infection^a^		
Yes	2.76 (1.05-7.19)	0.03
No		
HPV 16 and 18 infection		
Yes		
No	3.53(1.17-10.67)	0.02
Infection by HPV types other than HPV 16 and 18		
Yes		
No	2.25(0.80-6.36)	0.08

^a^Reference HPV-negative, CIN: Cervical intraepithelial neoplasia

## Discussion

Age at first sexual intercourse prior to 16 years of age was found to be significantly associated with HPV infection, particularly with high-risk oncogenic HPV 16 and 18 types, and with CIN 1 or worse in women referred because of an abnormal cervical smear. The diagnosis of CIN 1 or worse was also associated with HPV infection, particularly HPV types 16 and 18.

Several studies have shown that cervical infections occur shortly after sexual debut, emphasizing the importance of sexual intercourse in transmission [17]. The risk of infection also increases with each new sexual partner, underscoring the ease of transmission via sexual acts [17].

Women who begin to have sexual intercourse before the age of 16 are more vulnerable to HPV infection because during puberty the cervix undergoes cellular changes at the transformation zone that are known as ectopy [18]. During ectopy, the cervical cells may not only be more susceptible to HPV infection, but they may also be more prone to persistent HPV infection and to more lasting damage from an infection [19]. Almonte et al. [20] observed that having first sexual intercourse at an early age and having several sexual partners during a lifetime increased the risk of having high-risk HPV (age-adjusted odds ratio [AOR] for age at first sexual intercourse).

Early age at first sexual intercourse, which may be a marker for the total number of sexual partners, is also associated with the risk of progression of the disease among HPV-positive individuals [21]. An active HPV infection is likely to be dependent on recent sexual activity, hence acquired recently, whereas latent or persistent infection could have been influenced by past sexual behavior. A greater number of lifetime sexual partners may increase the risk of becoming infected with one or more HPV types over time [2].

In the present study, no association was found between the number of sexual partners and HPV infection or lesions of CIN 1 or worse. A study conducted in Latin America and the Caribbean showed that women tend to remain monogamous once married or cohabiting, while men do not [22]; therefore, a woman’s risk of contracting HPV infection depends to a great extent on the sexual behavior of her male partner(s).

Studies have consistently shown an increased risk of male genital HPV positivity with sexual behavior, which could lead to an increased risk of cervical cancer in their female partners. More studies are needed in order to increase understanding of the natural history of HPV infection in males and its relationship with the risk of acquiring HPV and the development of cervical cancer in women [8].

Cervical neoplasia associated with infection by individual HPV types has been evaluated up to a certain point and there are indications that the specific types lead to different risks with respect to persistence and progression [23]. HPV types 16 and 18 may account for around 70 % of all cervical cancer. HPV type 16 has the highest oncogenic potential, followed by type 18 as the next most virulent [1].

Significant associations were found between women who had their first sexual intercourse prior to 16 years of age and HPV 16 and HPV 18 infections and lesions of CIN 1 or worse. HPV 16 and 18 are the types most commonly associated with cervical cancer [11]. A recent report found a 10-year increased risk of cervical precancer and cancer for HPV 18 that was similar in magnitude to that found for HPV 16 [24].

Knowledge on the incidence of the different HPV genotypes is needed in order to focus appropriately on preventive, diagnostic and therapeutic measures. Mazarico et al. [25] showed that HPV-positive patients were younger (mean age 32.3 years) than HPV-negative women (39.8 years). Baandrup et al. [26] showed that HPV 16-associated CIN 3 lesions occur at a significantly younger age than lesions associated with other high-risk HPV infections.

This study showed that women who began to have sexual intercourse before the age of 16 had a 2.2-fold higher risk of developing CIN 1 lesions or worse. These data are in agreement with the findings of Flores et al. [27] who reported a two-fold higher risk of high grade CIN and cervical cancer in women who had their first sexual intercourse prior to 16 years of age. According to those authors, this increased risk may be attributable to age at first sexual intercourse since this event may be a proxy for the time of first HPV infection. Women who had sexual intercourse for the first time at a younger age may have been exposed to a persistent HPV infection for longer periods compared to women who began to have sex at a later age [28].

In relation to risk factors such as high parity, the use of oral contraceptives, number of pregnancies and smoking, no statistically significant association was found with HPV infection or lesions of CIN 1 or worse. Other investigators have also reported a negative association between HPV infection and these risk factors [11]. On the other hand, some studies have reported that the number of sexual partners, pregnancy and past or current smoking were all factors predictive of HPV infection [28–30].

In fact, the use of oral contraceptives and smoking were the two principal factors found to accelerate maturation and are also known to be important risk factors for cervical neoplasia [7, 31]. One limitation of the present study was the behavior profile of the women enrolled. The majority of women were not present or past smokers and this limitation precluded the possibility of carrying out any assessment on the effect of smoking on HPV acquisition.

As cervical cancer precursor lesions are the target of cervical cancer screening programs, it is important to improve understanding of the association between age and precancerous lesions of the cervix according to HPV type [26].

## Conclusions

Early events that can modify the carcinogenic potential of HPV infection include early age at first intercourse, which is a possible proxy of longer duration of persistent HPV infection. Understanding the epidemiology of genital HPV infection is important for the development of preventive actions against this infection and against HPV-related neoplasia.

## Abbreviations

HPV: Human papillomavirus
CIN: Cervical intraepithelial neoplasia
ICC: Invasive cervical carcinoma
